# Maternal obesity in pregnancy and children’s cardiac function and structure: A systematic review and meta-analysis of evidence from human studies

**DOI:** 10.1371/journal.pone.0275236

**Published:** 2022-11-08

**Authors:** Tamara den Harink, Manouck J. M. Roelofs, Jacqueline Limpens, Rebecca C. Painter, Tessa J. Roseboom, Arend W. van Deutekom

**Affiliations:** 1 Department of Epidemiology and Data Science, Amsterdam UMC, University of Amsterdam, Amsterdam, Netherlands; 2 Amsterdam Reproduction and Development, Amsterdam, The Netherlands; 3 Medical Library, Amsterdam UMC, University of Amsterdam, Amsterdam, The Netherlands; 4 Department of Obstetrics and Gynecology, Amsterdam UMC, Amsterdam Reproduction and Development, University of Amsterdam, Amsterdam, Netherlands; 5 Division of Paediatric Cardiology, Department of Paediatrics, Erasmus MC-Sophia Children’s Hospital, Rotterdam, The Netherlands; Finnish Institute for Health and Welfare: Terveyden ja hyvinvoinnin laitos, FINLAND

## Abstract

The prevalence of obesity is increasing worldwide. Experimental animal studies demonstrate that maternal obesity during pregnancy directly affects cardiac structure and function in their offspring, which could contribute to their increased cardiovascular disease (CVD) risk. Currently, a systematic overview of the available evidence regarding maternal obesity and alterations in cardiac structure and function in human offspring is lacking. We systematically searched the electronic databases Embase, MEDLINE and NARCIS from inception to June 29, 2022 including human studies comparing cardiac structure and function from fetal life onwards in offspring of women with and without obesity. The review protocol was registered with PROSPERO International Prospective Register of Systematic Reviews (identifier: CRD42019125071). Risk of bias was assessed using a modified Newcastle-Ottawa scale. Results were expressed using standardized mean differences (SMD). The search yielded 1589 unique publications, of which thirteen articles were included. Compared to offspring of women without obesity, fetuses of women with obesity had lower left ventricular strain, indicative of reduced systolic function, that persisted in infancy (SMD -2.4, 95% confidence interval (CI) -4.4 standard deviation (SD) to -0.4 SD during fetal life and SMD -1.0, 95% CI -1.6 SD to -0.3 SD in infancy). Furthermore, infants born to women with obesity had a thicker interventricular septum (SMD 0.6 SD, 95% CI 0.0 to 1.2 SD) than children born to women without obesity. In conclusion, cardiac structure and function differs between fetuses and children of women with and without obesity. Some of these differences were present in fetal life, persisted in childhood and are consistent with increased CVD risk. Long-term follow-up research is warranted, as studies in offspring of older age are lacking.

## Introduction

The prevalence of obesity is increasing worldwide [1,2], with some countries reporting up to half of women entering pregnancy with overweight or obesity [3]. Obesity before or during pregnancy is associated with adverse pregnancy outcomes, such as gestational diabetes mellitus (GDM), preeclampsia and preterm birth [4–7]. In addition, children born to women with obesity during pregnancy are more likely to develop obesity, type 2 diabetes and cardiovascular diseases (CVD) [8,9]. Furthermore, maternal obesity is associated with an increased risk of congenital heart disease in their children and premature death from cardiovascular events as compared to children born to women without obesity during pregnancy [10,11].

The exact pathophysiology of this increased CVD risk in offspring of women with obesity during pregnancy remains to be determined [9,10]. The increased CVD risk could be partially explained by the higher risk of hypertension and obesity observed in offspring of women with obesity [12,13]. However, experimental studies in animals demonstrate that maternal obesity during pregnancy directly affects cardiovascular development in their offspring which could also explain the increased CVD risk in the offspring [14–17]. For example, a mouse model demonstrated that maternal obesity during pregnancy resulted in systolic and diastolic dysfunction in the fetus, which persisted throughout adulthood and was independent of offspring’s body weight and postnatal diet [17]. In addition, offspring born to obese mice and sheep demonstrated cardiac hypertrophy and fibrosis [14,15].

To our knowledge, no systematic review has addressed the relation of maternal obesity in humans and cardiac alterations in their offspring, excluding congenital heart disease. We therefore conducted a systematic review on the available evidence on this topic.

## Methods

We performed a systematic review according to the Preferred Reporting Items for Systematic Reviews and Meta-Analyses guidelines. The review protocol was registered with PROSPERO International Prospective Register of Systematic Reviews (identifier: CRD42019125071, first version on April 12^th^ 2019, updated version February 10^th^ 2021). Ethical approval: This article does not contain any studies with human participants performed by any of the authors.

### Search strategy

A medical information specialist (JL) performed a systematic search in OVID MEDLINE, OVID EMBASE and NARCIS (scholarly information in the Netherlands) from inception to June 29, 2022. Search terms included controlled terms (i.e. MeSH-terms in MEDLINE) and free text terms for the following concepts: [1] obesity/ weight gain; [2] (a) fetal heart, fetal programming or prenatal exposure or (b) (pre)-pregnancy and offspring and [3] heart function or structure. Animal studies were excluded. No other restrictions, including date and language restrictions, were applied. For the complete search strategy, see S1 Table. We additionally searched the reference lists of included papers and the papers citing these studies using Web of Science for additional relevant publications. Citations were imported and deduplicated using EndNote® [18].

Two reviewers (TdH and MR) independently screened titles and abstracts for eligibility using Rayyan as a web tool (http://rayyan.qcri.org). Disagreements were resolved through discussion with a third reviewer (AvD) until consensus was reached. The full texts of relevant articles were screened for eligibility. If full texts were not available through the library system, we contacted authors directly to request full texts. Full text screening was done by the same two independent reviewers.

### Inclusion and exclusion criteria

Studies were eligible if they:

- reported on cardiac function or structure as measured by echocardiography or magnetic resonance imaging (MRI) *and*
- reported on the outcomes of fetuses and offspring from mothers with maternal obesity, defined as body mass index (BMI) ≥ 30 kg/m^2^ before and/or during pregnancy *and*
- reported on the outcomes of fetuses and offspring of control pregnancies with a BMI <30 kg/m^2^ before and/or during pregnancy

Preconception BMI was defined BMI measured within 6 months before pregnancy. Conference abstracts were included only if the contained enough data relevant to the outcomes of interest and to adequately assess risk of bias.

We excluded studies if 1) It was not possible to differentiate between the outcomes of women with obesity and those of controls, 2) they focused on the incidence of congenital heart disease in offspring, or 3) they included women exclusively based on their higher GDM risk.

### Outcomes

Primary outcomes were determined on the basis of clinical utility and validity of the measures according to the American Society of Echocardiography Pediatric and Congenital Heart Disease Council [19]: (1) markers of left ventricle (LV) structure and dimension, including interventricular septum diameter at end diastole (IVSd), left ventricular internal diastolic diameter (LVIDd), end-diastolic left ventricular posterior wall thickness, left ventricle mass (LVM), LVM indexed for body surface area (BSA) (LVMI), relative wall thickness, end-diastolic volume indexed for BSA (EDVi) and end-systolic volume indexed for BSA (ESVi), (2) markers of systolic function, including shortening fraction (SF), ejection fraction (EF), tissue Doppler derived peak systolic velocity (s’), longitudinal strain (LS) and tricuspid annular plane systolic excursion (TAPSE), (3) markers of diastolic function, including isovolumic relaxation time, mitral valve E/A ratio, tissue Doppler derived early (e’) and late (a’) diastolic velocity, and (4) global cardiac functioning as expressed with the myocardial performance index. We included LS measurements derived from the apical 4-chamber view, or all apical views (2-, 3- and 4-chamber). LS has a negative value, but we will refer to lower strain as a value closer to zero, meaning reduced systolic function. Other echocardiographic outcomes and MRI derived parameters were assessed as secondary outcomes.

### Data extraction and quality assessment

Data extraction was performed by two independent reviewers (TdH and MR). We stratified the cardiac outcomes for the following developmental stages: (1) fetuses, (2) neonates (< 28 days of age) (3) infants (28 days to 1 year of age), (4) children (1 to 12 years), (5) adolescents (12 to 18 years) and (6) adults (>18 years). For each included study the following parameters were collected: (1) study design, (2) definition of maternal obesity, (3) timing of maternal BMI measurement, (4) fetal/offspring’s age at outcome assessment, (5) number of participants and (6) relevant outcomes, including the numbers, mean/standard deviation (SD) for normally distributed variables, and median/range for variables that were not normally distributed. We also collected information on potential confounders, including: prevalence of type 1 and type 2 diabetes, GDM and hypertensive disorders of pregnancy, gestational age, birthweight, sex, maternal age, offspring blood pressure and offspring heart rate. Since cardiac mass and dimensions in the pediatric population are usually adjusted for BSA or weight we also collected offspring anthropometrics at time of measurement [20]. In case of fetal studies, maternal blood pressure and heart rate was collected if available. If, in addition to groups with and without obesity, a study consisted of a third group of women with type 1 or 2 diabetes or GDM, we excluded this/ these group (s). If not all required data was present in the full text, we contacted the authors for additional data. If maternal BMI followed a normal distribution and was categorized in more than two groups in the original paper, we calculated the pooled means and SD to create a group with obesity (BMI ≥30 kg/m^2^) and a control group (BMI <25 kg/m^2^) group where possible. If only the 95% CI for normally distributed variables was available, SD was calculated manually.

For the assessment of the methodological quality of the articles two independent reviewers (TdH and MR) used the Newcastle-Ottawa Scale (NOS) for cohort studies [21]. Studies were assessed on three categories; selection, comparability and outcome. For cross-sectional studies, we used the NOS for cohort studies excluding the assessment of the follow-up period. S4 and S5 Figs demonstrate our adjusted NOS risk of bias form for cohort and case-control studies, respectively. A maximum of 9 or 7 stars could be awarded to cohort and cross-sectional studies, respectively. Low risk of bias was defined as a final score of 8–9 or 6–7 stars, moderate risk of bias was given for 7 or 5 stars and high risk of bias for 6 or 4 stars or less for cohort and cross-sectional studies, respectively. Funnel plots were used to assess possible publication bias in outcomes that included 10 or more studies [22].

### Statistical analyses

#### Meta-analyses

Two or more articles reporting on the same cardiac outcome in the same developmental stage were included for pooled analyses using Cochrane Collaborations RevMan Software version 5.4 (The Cochrane Collaboration, Copenhagen, Denmark) [22]. If one article reported on repeated measurements at different developmental stages, we included the measurement that best matched the other studies for meta-analyses. Meta-analyses were performed using a random effects model. Due to differences in methods of assessment, for example strain being measured only in four-chamber view, or both two- and four-chamber view, we reported standardized mean differences (SMD) with 95% confidence intervals (CI). We defined low and high heterogeneity according to I^2^ cut-offs of 30% and 75%, respectively [23]. Articles were not excluded in our meta-analyses due to high heterogeneity, but potential sources causing high heterogeneity were discussed.

Outcomes that could not be included in our meta-analyses due to inability to extract a 2x2 table or single measurements were described narratively. If the same cardiac outcome measure was described at different ages, either within one article or in different articles, we constructed boxplots for a visual representation of the development over time. In our boxplots we plotted the SMD of cardiac outcomes against time. The SMD and 95% CI were derived from single measurements or when available, from our meta-analyses.

#### Sensitivity and subgroup analyses

Maternal obesity is often accompanied by pregnancy induced hypertension and hyperglycemia in pregnancy [24,25]. These comorbidities are independently associated with alterations in offspring’s cardiac outcomes [26,27]. To evaluate if cardiac outcomes in offspring of women with obesity before or during pregnancy are independent from maternal hypertension and maternal glucose regulation disorders we performed sensitivity analyses excluding articles that included women with type 1 or 2 diabetes, GDM or hypertensive disorders of pregnancy. We also performed subgroup analyses exploring possible sex differences in the outcomes.

## Results

### Description of included studies

**Fig 1:**
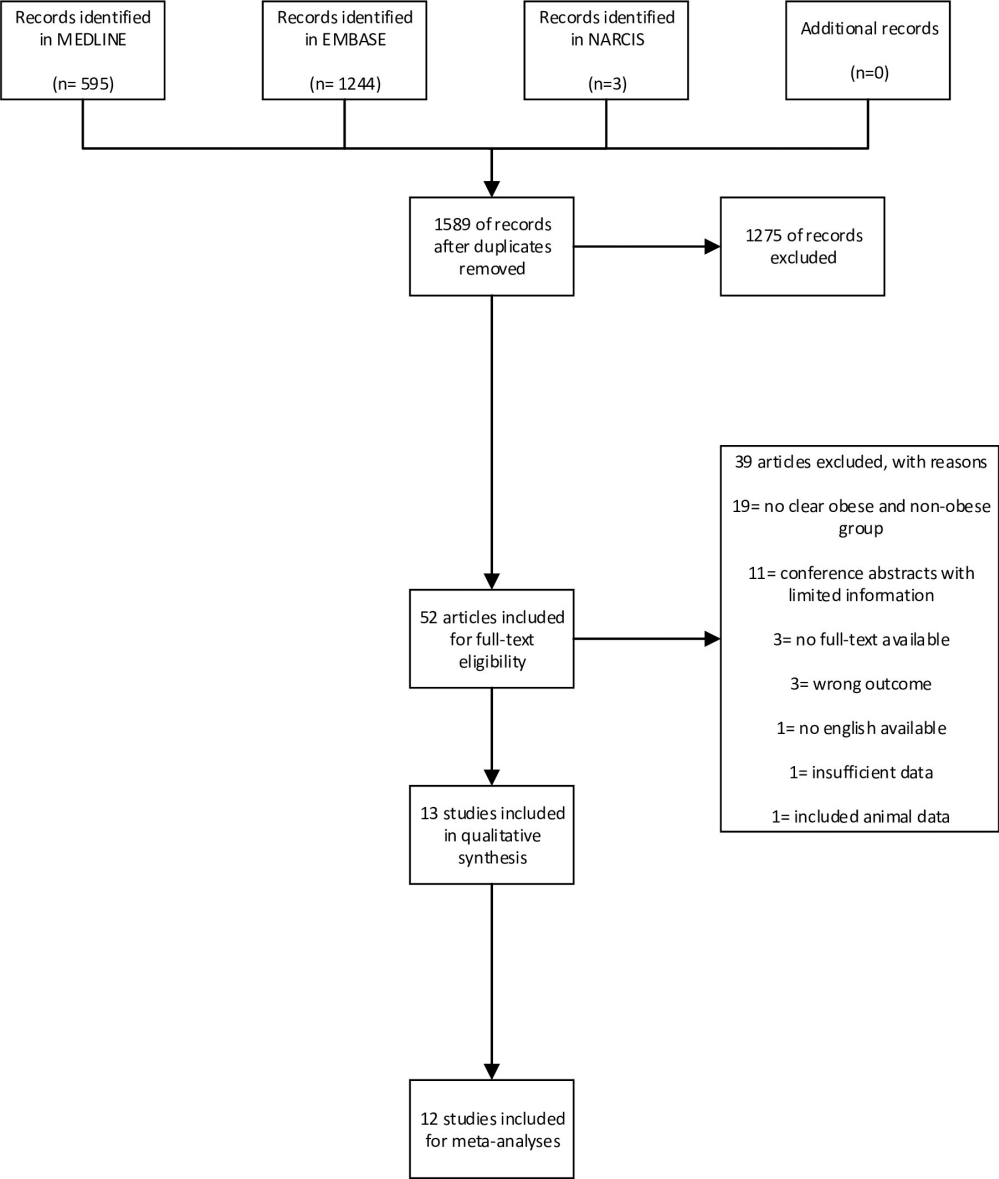
PRISMA flowchart.

The literature search identified 1589 unique publications (see Fig 1). After title and abstract and full-text screening, thirteen articles were included. For the rationale and reasons for exclusions, see Fig 1. These thirteen articles contained data from ten original studies, comprising offspring of 1068 women with and 7615 women without obesity before or during pregnancy. Ten articles included data measured by echocardiography [28–38], and two articles reported on data measured by MRI [39,40].

Table 1 shows the characteristics of the included studies. Six articles reported on outcomes in fetuses (n = 1483) [28–33], three articles on outcomes in neonates (n = 187) [34,35,39], three on outcomes in infants (children <1 year of age) (n = 234) [34–36] and three articles reported on outcomes in children older than 1 year of age (n = 6966) [38,40,41]. No articles reported on cardiac structure or function in adolescents or adults born to women with obesity. S2 Table demonstrates the outcomes measured per included study.

**Table 1:** Characteristics of included studies.

Developmental stage	Study	Type of study	BMI thresholds		Moment of BMI measurement	Age child at follow up	DM I & II /GDM		Hypertensive pregnancies		N	
			Group with obesity	Control			Group with obesity	Control	Group with obesity	Control	Group with obesity	Control
**Fetal**	Ali 2020^%^	Cross-sectional	≥ 30 kg/m^2^	<30 kg/m^2^	20–35 weeks GA	20–35 weeks GA	No DM I/II		PE: None		183	838
							Unknown GDM		HT: no description			
	Bayoumy 2020	Cohort	≥ 30 kg/m^2^	≤ 25 kg/m^2^	Preconception	30 weeks GA	No DM I/II & GDM		Unknown		30	25
	Ece 2014	Cross-sectional	≥ 30 kg/m^2^	19–25 kg/m^2^	Preconception	+/- 32 weeks GA	No DM I/II & GDM		No pre-eclampsia or hypertension		54	44
	Ingul 2016^^,^*	Cohort	≥ 30 kg/m^2^	≤ 25 kg/m^2^	Preconception	14 weeks GA	DM II: 3/49 (6.1) GDM: 3/49 (6.1)	None	HT: 2/52 (3.8)	None	49	23
						20 weeks GA		PE: 2/52 (3.8)				
						32 weeks GA						
	Kulkarni 2017^$^	Cross-sectional	≥ 30 kg/m^2^	<30 kg/m^2^	25 weeks GA	+/- 25 weeks GA	No DM I/II & GDM		HT: 5/26 (19.2)	No description	26	70
	Lee-Tannock 2021	Cohort	≥ 30 kg/m^2^	<25 kg/m^2^	No description	18–20 weeks GA	Unknown		Unknown		43	98
						20–24 weeks GA						
						24–28 weeks GA						
						32–36 weeks GA						
						36–40 weeks GA						
**Neontal/Infant**	Groves 2021	Cohort	≥ 30 kg/m^2^	20–25 kg/m^2^	First trimester	<3 days	No DM I/II & GDM		No description		31	56
	Cade 2017^$^	Cohort	30–45 kg/m^2^	<30 kg/m^2^	Preconception	1 month	No DM I/II & GDM		No description		24	23
	Guzzardi 2018^%^^,^^	Cohort	≥ 30 kg/m^2^	<30 kg/m^2^	Preconception	Birth	No DM I/II GDM: 44.4%	No DM I/II GDM: 22.2%	No description		9	43
						3 months						
						6 months						
						12 months						
	Nyrnes 2018^^,^*	Cohort	≥ 28 kg/m^2^	18.5–25 kg/m^2^	Preconception	1–3 days	GDM: 7/28 (25)	0	No description		28	20
						6–8 weeks						
**Children**	Santos 2019^#^	Cohort	≥ 30 kg/m^2^	<25 kg/m^2^	Preconception	10 years	Unknown		No description		167	2187
	Toemen 2016^#^	Cohort	≥ 30 kg/m^2^	<25 kg/m^2^	Preconception	6 years	GDM: 13/396 (3.2)	GDM: 15/3400 (0.4)	No description		402	3508
	Wang 2021	Cohort	≥ 30 kg/m^2^	20–25 kg/m^2^	Preconception	4 years	5 (22.7)*	107 (15.7)*	No description		22	680

*&^#^ consisted partly of the same subjects.

^%^ Unpublished data.

^$^ Study included women with (pre)gestational diabetes. However these were categorized in another group and therefore not included in the maternal obesity group.

*Unknown which type of diabetes.

BMI = Body mass index.

GA = Gestational age.

DM = Diabetes Mellitus.

GDM = gestational DM.

HT = hypertension.

PE = pre-eclampsia.

Four articles reported on repeated measures [30,33–35]. Ingul *et al*. [30] included fetal cardiac outcomes at 14, 20 and 32 weeks of gestation. Lee-Tannock *et al*. [33] described cardiac measurements in fetal life every four weeks from inclusion until delivery. Guzzardi *et al*. [34] reported on cardiac outcomes in neonates and infants, including measurements at birth and 3, 6 and 12 months of age. The article of Nyrnes *et al*. [35] consisted of partly the same cohort as Ingul *et al*., but reported on cardiac outcomes measured at 1–3 days and 6–8 weeks after birth. To best match the other included studies, data measured at 32 weeks of gestation by Ingul *et al*. and 28–32 weeks of gestation by Lee-Tannock *et al*. were used in our meta-analyses on fetal outcomes. For the neonatal stage, we included outcomes at birth from Guzzardi *et al*. and 1–3 days of age from Nyrnes *et al*. Outcomes at 3 months of age from Guzzardi *et al*. and outcomes at 6–8 weeks of age from Nyrnes *et al*. were included in the infant stage.

### Cardiac structure

Meta-analyses for cardiac structure were possible for IVSd in fetal life and during infancy, EDVi and ESVi in the neonatal stage, LVMI in the neonatal, infancy and childhood stage and relative wall thickness in childhood (see Table 2 and S1 Fig for forest plots). IVSd did not differ in fetuses from women with obesity as compared to controls (SMD 0.1, 95%CI -0.43, 0.70). However, two studies showed that during infancy IVSd was increased in infants born to women with obesity as compared to controls (SMD 0.6, 95% CI 0.04, 1.19). LVMI did not differ between neonates, infants and children born to women with or without obesity before or during pregnancy (SMD -0.1 95% CI -0.79, 0.61, SMD 0, 95% CI -0.80, 0.79 and SMD 0.22, 95% CI -0.02, 0.45, respectively). EDVi and ESVi in the neonatal stage did not differ between those born to women with and without obesity (SMD -1.9, 95% CI -4.09, 0.30 and SMD 0.0 95% CI -1.09, 1.05, respectively). Relative wall thickness was not significantly different between children born to women with and without obesity (SMD 0.34, 95% CI -0.38, 1.07).

**Table 2:** Summary of meta-analyses for primary outcomes of cardiac structure and function in offspring born to women with obesity compared to offspring of control group results are presented as standardized mean difference (SMD) [95% confidence interval].

	Number of studies	Number of participants included		Fetal	Neonatal	Infant	Children
		Maternal obesity	Control	SMD^ (95% CI)	SMD^ (95% CI)	SMD^ (95% CI)	SMD^ (95% CI)
IVSd	4	139	163	0.1 [-0.43, 0.70]		0.6 [0.04, 1.19]*	
LVMI	4	33	66		-0.1 [-0.79, 0.61]	0.0 [-0.80, 0.79]	0.22 [-0.02, 0.45)
EDVi	2				-1.9 [-4.09, 0.30]		
ESVi	2				0.0 [-1.09, 1.05]		
RWT	2						0.34 [-0.38, 1.07]
TAPSE	4	176	190	-1.2 [-2.30, 0.01]			
LV global strain	5	157	161	-2.4 [-4.42, -0.36]*		-1.0 [-1.56, -0.33]*	
RV global strain	2	52	43			-1.1 [-2.83, 0.63]	
EF	3	33	66		0.1 [-0.40, 0.68]	0.6 [-0.58, 1.68]	
SF	2	52	43			0.2 [-1.23, 1.54]	
LV e’	3	133	92	-0.4 [-1.52, 0.78]			
LV a’	3	133	92	-1.5 [-4.01, 0.97]			
IVS e’	2	103	67	0.2 [-0.16, 0.46]			
IVS a’	2	103	67	0.5 [-0.52, 1.47]			
MPI	3	263	952	0.5 [-0.03, 0.96]			
MV e/a	3	129	137	0.0 [-0.28, 0.23]			
IVRT	2	80	114	1.6 [-6.42, 9.65]			

* = p<0.05.

^results presented are not corrected for confounding variables.

IVSd = Interventricular septum at end-diastole.

LVMI = Left ventricular mass index.

EDVi = LV end diastolic volume indexed for BSA.

ESVi = LV end systolic volume indexed for BSA.

RWT = relative wall thickness.

TAPSE = Tricuspid annular plane systolic excursion.

LV = Left ventricle.

RV = Right ventricle.

EF = Ejection fraction.

SF = Shortening fraction.

SV = Stroke volume.

MV = Mitral valve.

MPI = Myocardial performance index.

IVRT = Isovolumic relaxation time.

**Fig 2:**
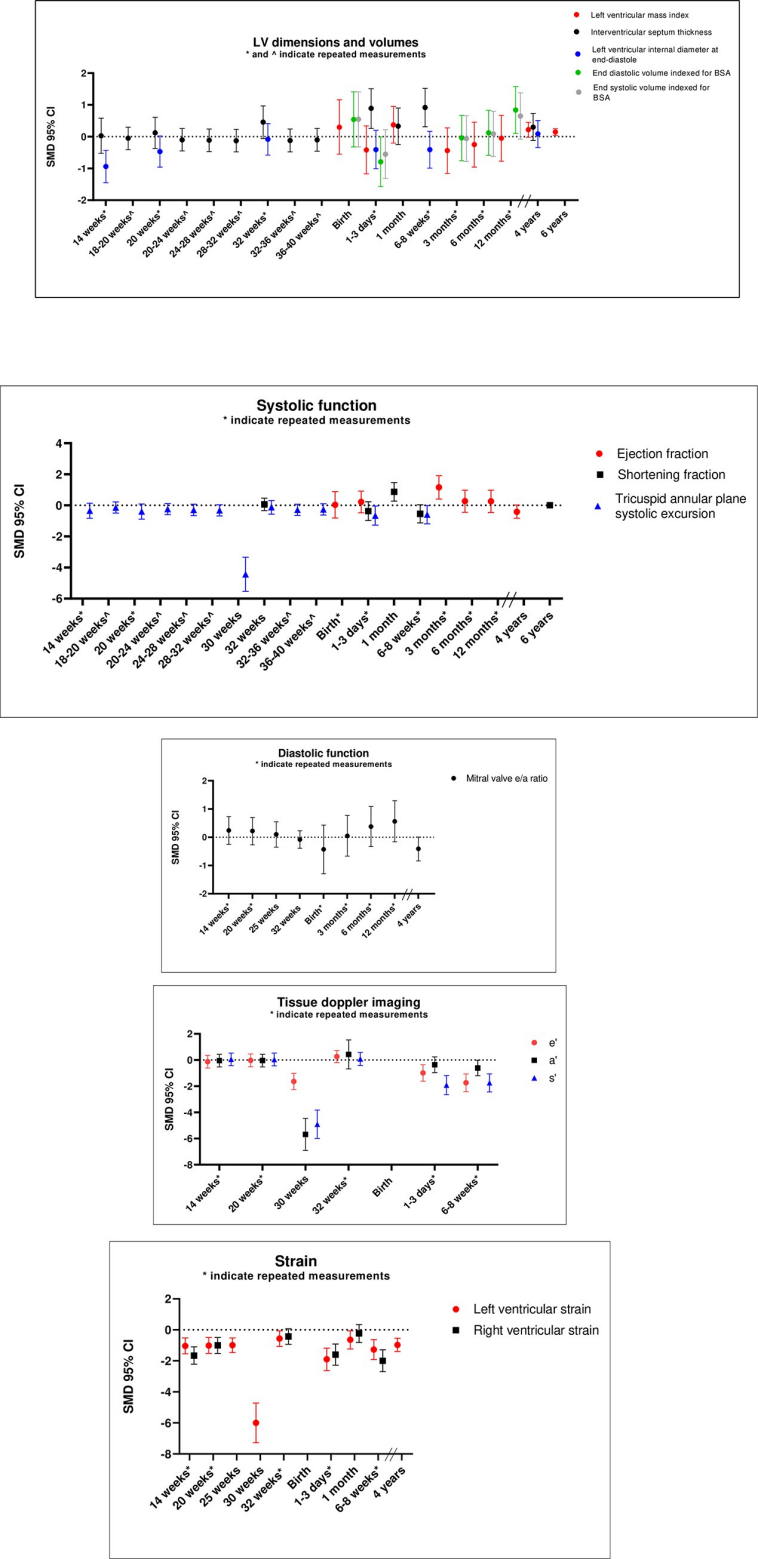
Boxplots. a: Boxplots demonstrating associations between maternal obesity and cardíac alterations in offspring measured at múltiple timepoints in different individuals, as expressed by standard mean deviations (SMD) and 95% confidence intervals (Cl). b: Boxplots demonstrating associations between maternal obesity and cardíac alterations in offspring measured at múltiple timepoints in different individuals, as expressed by standard mean deviations (SMD) and 95% confidence intervals (Cl).

Cardiac structural outcomes not available for meta-analyses were displayed in boxplots. Boxplots for the association of offspring’s IVSd, EDVi, ESVi, LVMI and LVIDd with maternal obesity through different developmental periods are displayed in Fig 2. Fetuses of mothers with obesity had a lower LVIDd at 14 weeks as compared to controls, but this difference disappeared later in life. A single study demonstrated that, at 6 years of age, children born to women with obesity have a significantly higher LVMI as compared to controls. Another study demonstrated a significantly thicker left ventricular wall thickness at end-diastole in children born to women with obesity as compared to controls (Table 3).

**Table 3:** Primary outcomes available in single studies born to women with obesity vs born to women without obesity data presented as standardized mean difference [95% CI] unless stated otherwise.

	Fetal	Infants	Children
RWT		0.1 [0.01 to 0.11]*	
LVPWd		-0.3 [-0.97, 0.47]	0.62 [0.19, 1.04]*
ET	-0.1 [-0.47, 0.32]		
IVCT	0.0 [-0.36, 0.43]		

* = p<0.05.

RWT = Relative wall thickness.

LVPWd = Left ventricular posterior wall thickness.

ET = Ejection time.

IVCT = Isovolumic contraction time.

Single studies reported no associations between end-diastolic left ventricular posterior wall thickness in infants and maternal obesity. Relative wall thickness was higher in infants born to women with obesity as compared to controls, but this difference was not visible in children. Kulkarni *et al*. [32] reported on non-normally distributed data in fetuses and demonstrated that obesity before or during pregnancy was not associated with increased IVSd (fetuses of mothers with obesity: median 2.0, interquartile range (IQR) 1.6–2.9; fetuses of lean mothers: median 1.9, IQR 1.4–2.5) [32].

### Systolic function

Meta-analyses for systolic function demonstrated that maternal obesity was associated with lower LV strain in fetal life (SMD -2.4, 95% CI -4.42, -0.36) and infancy (SMD -1.0, 95% CI -1.56, -0.33) as compared to controls. RV strain, EF, SF and TAPSE were not significantly different between neonates or infants born to women with and without obesity before or during pregnancy (Table 2 and S2 Fig).

Boxplots were available for LV strain, RV strain, SF, EF, TAPSE and LV s’ (Fig 2). LV strain in fetuses and offspring of women with obesity was consistently lower at every measured time point, ranging from 14 weeks of gestation until 4 years after birth. RV strain was also lower in fetuses and neonates of women with obesity as compared to controls at almost all time points (Fig 2). Fetuses and infants of women with obesity demonstrated significant lower LV s’ and TAPSE at a few time points as compared to controls (see Fig 2). Kulkarni *et al*. reported on non-normally distributed data and demonstrated a significantly higher EF in fetuses of women with obesity as compared to fetuses of women without obesity (median 60%, range 55 to 66; median 68%, range 61 to 76, respectively, p = 0.01). No significant differences in single studies were found for ejection time and isovolumic contraction time (Table 3).

### Diastolic and general function

In fetal life, no associations between maternal obesity and LV e’ or a’ were found (Table 2). In addition, isovolumic relaxation time, MV E/A and myocardial performance index did not differ between fetuses from mothers with obesity as compared to controls (Table 2 and S3 Fig for forest plots). The association between LV e’, a’ and MV E/A with maternal obesity at every measured time point were also demonstrated in a boxplot (Fig 2). The difference in LV e’ and a’ between infants born to women with obesity and controls seemed to increase after birth, with infants born to women with obesity demonstrating lower velocities. This difference was most pronounced for LV e’ velocity. MV e/a did not seem to differ between fetuses and infants of women with obesity and their controls.

### Secondary outcomes

Santos *et al*. [40] found that, at age 10, children born to women with obesity had a higher pericardial fat mass as measured with MRI indexed to height as compared to children born to lean women and women with underweight (median 13.3 g, 95% CI 5.5, 25.1 and median 10.4 g, 95% CI 4.4, 21.9, respectively).

### Quality assessment

Thirty eight percent of included studies had low risk of bias, 8% had moderate risk of bias and 54% had high risk of bias (see S6 and S7 Figs). Most bias occurred due to self-reporting of weight, making blinding for maternal BMI status during fetal image acquisition impossible. All of our meta-analyses demonstrated considerable heterogeneity (see S1–S3 Figs). Due to the low number of studies included, funnel plots were deemed not appropriate and publication bias could not be assessed.

### Sensitivity and subgroup analysis

Five of thirteen (39%) articles excluded women with GDM. Two studies included women with diabetes, but the incidence of type 1 or 2 diabetes and GDM was not mentioned among women with obesity or controls [33,41]. Two other articles did not mention type 1 or 2 diabetes and GDM [31,40]. In the remaining studies, the prevalence of GDM ranged from 3.2% to 44.4% among women with obesity. Three articles (30%) described the prevalence of hypertensive pregnancies and/or pre-eclampsia. In summary, subgroup analyses to assess the mediating effect of type 1 or 2 diabetes, GDM or preeclampsia in the association of maternal obesity with offspring cardiac outcomes were not feasible as a result of insufficient data.

Eight articles (62%) reported offspring sex, but none mentioned sex specific effect sizes on cardiac outcomes. Therefore, we could not explore possible sex differences in our meta-analyses.

Studies including infants did not always mention anthropometric measures at time of echo which could potentially have affected cardiac structure outcomes in our meta-analyses. IVSd was measured in the study of Cade *et al*. [36] and Nyrnes *et al*. [35], where the latter found no statistical difference in weight at time of echo between infants born to women with obesity and controls. The study of Cade *et al*., including neonates one month after birth, did not mention body weight at time of echo. However, no significant difference in birthweight between neonates born to women with obesity and controls was found.

## Discussion

In this systematic review and meta-analysis of thirteen studies including 1068 fetuses and offspring of women with obesity before or during pregnancy and 7615 controls, we found evidence that cardiac structure and function differs between fetuses and children of women with obesity and those born to women without obesity. Some of these differences were already present in fetal life and persisted throughout childhood. LV strain was lower in fetuses of women with obesity and persisted after birth, indicating reduced cardiac function as compared to offspring of women with normal weight. There was also evidence of structural cardiac changes related to maternal obesity, as infants of mothers with obesity showed an increased IVSd as compared to controls. Since impaired strain and increased IVSd are associated with an increased CVD risk in later life [42,43], these alterations could contribute to the increased CVD risk observed among offspring of women with obesity. However, data regarding the association between maternal obesity and cardiac alterations beyond childhood are lacking, so how these maternal obesity-associated alterations in cardiac structure and function relate to future CVD risk should be the aim of future research.

We found an association between maternal obesity and increased IVSd in infancy. Several epidemiological studies have found that structural cardiac changes in young adults are predictive of future CVD events [44]. For example, in healthy young adults, increased IVSd is independently associated with an increased future risk for hypertension [43,45]. Although these associations of cardiac structure with future CVD risk have not been described in children, it is known that markers of cardiac structure track throughout childhood to adolescence and beyond [46,47]. Therefore, the altered cardiac structures we found in children of women with obesity might persist to adulthood and provide an explanation for the increased CVD risk in offspring of women with obesity.

Strain measurements describe the deformation of the heart during the cardiac cycle and provide important information on cardiac function [48]. In a low risk-population, cardiac strain measurements predict long-term risk of cardiovascular morbidity and mortality and are therefore important markers of cardiac health [49]. Although strain is a reliable measure of fetal cardiac function, assessing strain in fetuses is challenging due to the small size of the fetal heart and the high fetal heart rate [50]. In addition, in women with obesity, fetal strain measurements could be compromised due to the limitations of visualizing fetal structures caused by maternal abdominal subcutaneous adipose tissue [51]. However, the studies included in our meta-analyses attempted to increase image reliability, either by demonstrating moderate agreement in the interobserver analyses, excluding measurements that failed to be tracked or by taking the mean strain in three consecutive cycles. Strain values are known to track throughout childhood, suggesting that the impaired strain associated with maternal obesity in fetal life and infancy might track to childhood and beyond and explain the increased CVD risk in offspring of mothers with obesity [52]. We did not find other signs of systolic dysfunction in offspring born to women with obesity. However, animal studies have also described systolic dysfunction in offspring born to obese dams [17]. Therefore, we hypothesize that maternal obesity is indeed a risk factor for systolic dysfunction in their children. However, this finding must be validated in future larger studies.

Although animal studies found diastolic dysfunction in fetuses of obese animals due to cardiac fibrosis and the consequent reduction of ventricular compliance [14], we did not find signs of diastolic dysfunction in fetuses of women with obesity as compared to their controls. However, single studies demonstrated lower LV e’ and a’ velocities in fetuses and neonates born to women with obesity as compared to controls, indicating impaired diastolic function (Fig 2).

### Effect of offspring’s age on maternal obesity associated cardiac differences

We found an association of maternal obesity with higher IVSd during infancy, but not in fetal life. This is in contrast to studies assessing the association of GDM and cardiac alterations in offspring, where maternal hyperglycemia in utero and the resulting fetal hyperinsulinemia, leads to myocardial hypertrophy [27], which gradually normalizes after birth [53]. The obesogenic pregnancy is characterized by lower glucose levels and different hemodynamic and metabolic effects during fetal life as compared to diabetic pregnancies, which could explain the different trajectories. Also, measurement of fetal IVSd is sometimes complex due to the position of the fetus and its small heart dimensions and the suboptimal views caused by maternal abdominal subcutaneous adipose tissue [51,54]. Therefore, the lack of a difference in IVSd in fetuses of women with obesity and their controls might be due to measurement error and small size of the study groups.

Our boxplots demonstrated differences in cardiac structure and function of fetuses and children born to women with obesity as compared to their controls (Fig 2A and 2B). Although these differences did not all reach statistical significance, there was a clear trend towards inferior cardiac structure and function in children born to women with obesity. The study with the oldest children [38] demonstrated that children born after maternal obesity had a significantly higher LVMI but no difference in SF (not corrected for childhood’s BMI) as compared to their controls at 6 years (Fig 2A) [38]. This suggests that there is a sustained effect of maternal obesity on cardiac alterations in their children. In a mouse model, cardiac hypertrophy in offspring of obese rodents was hypothesized to act as a protective mechanism for cardiac dysfunction [17]. Blackmore *et al*. postulate that the cardiac hypertrophy eventually subsides due to inadequate cardiac function and therefore inability to provide for the protective mechanism. We hypothesize that LVMI increases gradually in children born to women with obesity. This could be the result of a protective mechanism to compensate for subclinical cardiac systolic functional impairment as demonstrated with impaired longitudinal strain.

Unfortunately, no data was available on cardiac alterations in adolescents and adults exposed to maternal obesity during pregnancy. To determine if the associations of maternal obesity during pregnancy and cardiac alterations in their offspring are sustained throughout life, longer follow-up studies are warranted, preferably including longitudinal assessments of cardiac structure and function in children born to women with obesity and their controls.

### Underlying mechanisms for increased CVD risk in children born to women with obesity

Obesity during pregnancy is associated with increased blood pressure and obesity in children, which are factors known to influence cardiac structure and function [13,55,56]. However, Blackmore *et al*. demonstrated that cardiac dysfunction in mice born to obese dams preceded changes in body weight, indicating that cardiac dysfunction in offspring occurs independent of body weight [17]. In humans, studies in healthy children demonstrated that increased cardiac mass was not, or only to a very limited degree, related to increased blood pressure [57,58]. This suggests that the association of maternal obesity with offspring’s cardiac structure and function is at least partly independent of offspring’s blood pressure and body weight. We could not sufficiently test this hypothesis in our meta-analyses, because few studies reported analyses adjusted for blood pressure and weight of children (S3 Table).

Maternal obesity is associated with an increased risk of GDM which has previously been associated with cardiac alterations in fetuses [27,59]. Therefore, it is likely that some of the effect of maternal obesity on cardiac alterations in their offspring is mediated by maternal glycemic dysregulation. However, cardiac alterations in children born to women with diabetes have previously been described as transient [53]. We found that LVMI was higher in children aged 6 years born to women with obesity as compared to controls. Previous research did not find a significant difference in LVMI in children born to women with and without GDM [60]. In women with obesity a wide range of metabolic abnormalities are present in addition to glycemic dysregulation. Elevated leptin, insulin and lipid levels are features of obesity, each of which might also contribute to differences in cardiovascular development in the next generation [61,62]. An increase in these biochemical factors is known to induce impaired smooth cell proliferation, which can impair angiogenesis, vasoconstriction and increased platelet aggregation [63–65]. Together with the inflammatory state common to obesity, which can impair placental development and function and result in decreased blood flow to the fetus resulting in aberrant fetal cardiac function and development [63–65]. As a result, this could lead to significant hemodynamic changes in the fetal circulation in order to maintain the cardiac output, which could also provide an explanation for the differences found during fetal life [66].

Alternatively, the cardiac alterations described in this review could be transient and not responsible for the increased CVD risk in children born to women with obesity. Several other mechanisms have been described that could also explain the increased CVD risk in children born to women with obesity. Epigenetic modifications caused by adverse prenatal environment may be a possible mechanism underlying fetal programming of CVD [67,68]. Myocardial miRNAs expression (small RNA molecules involved in regulation of cellular processes such as proliferation, cell death and fibrosis) have been demonstrated to differ in fetuses of obese baboons as compared to fetuses of normal weight baboons [67]. Interestingly, the affected miRNAs have been associated with cardiac hypertrophy and enhanced fibrosis. In children, maternal obesity is also associated with altered DNA methylation [69,70]. This is demonstrated in a sibling study showing different methylation patterns of genes associated with improved cardiometabolic health in children born after their mother underwent bariatric surgery, as compared to their siblings born before bariatric surgery [71]. This suggests that epigenetic modifications could be a possible underlying mechanism responsible for the increased CVD risk in children born to women with obesity during pregnancy.

### Limitations

Our results should be interpreted within the framework of its inherent limitations. First, due to the observational design of the included studies, we cannot draw conclusions regarding causality between maternal obesity and offspring’s cardiac structure or function. Second, due to the small number of studies, we could not include more than three articles per meta-analysis, therefore not all primary outcomes could be evaluated at an aggregated level. We found no data on adolescent and adult offspring, making our conclusions less robust. Third, we could not investigate the mediating effects of GDM, hypertension during pregnancy, body size and offspring’s sex due to limited availability of such information in the studies included. Fourth, our risk of bias assessment demonstrated high risk of bias in 54% of the included studies. This was largely due to group allocation based on self-reported BMI, which is usually underestimated in populations with obesity (and overestimated in the lower ranges of BMI). Furthermore, studies did not always clearly report if the data analysis was carried out blinded. Fifth, there was high heterogeneity between studies (S1–S3 Figs). This could be the result of differences in gestational age at birth, maternal age at inclusion and offspring blood pressure. Unfortunately, few studies reported on these variables (S3 Table), which precludes the assessment of the cause of this heterogeneity. The study of Bayoumy *et al*. [29] demonstrated a significantly lower SMD in LV strain at 30 weeks of age as compared to other fetal studies. This was due to very small standard deviations of the outcome parameters presented in the article, with as a result a high standardized mean difference. If we exclude this ‘outlier’ from our meta-analyses, the difference in LV strain between fetuses from women with and without obesity is smaller, but remains statistically significant, suggesting the results found in our meta-analyses are robust. Furthermore, overweight women (i.e., women with a BMI 25-30kg/m^2^) were sometimes included in the control group, depending on the study’s definition of cases and controls. This could impair the discriminative power of the studies, as offspring in the control group might also experience a suboptimal perinatal environment. Therefore, our results must be interpreted with care and be replicated in future studies. Last, some articles measured women’s BMI during the second half of pregnancy. A maternal BMI ≥ 30 kg/m2 in this period might reflect gestation-related weight gain and does not necessarily reflect maternal preconception obesity. This could have resulted in women incorrectly being identified as having obesity.

### Conclusions

Children of women with obesity during pregnancy have signs of reduced cardiac function as compared to children of women without obesity. In addition, children born to women with obesity have increased IVSd as compared to controls. Since these structural and functional cardiac changes are found to be associated with increased susceptibility to CVD in later life, this could (partly) explain the increased CVD risk in children born to women with obesity. However, current literature on the association of maternal obesity on cardiac structure and function in offspring is sparse and limited to fetuses, infants, and young children. This highlights the need for long-term follow-up studies assessing the association of maternal obesity and cardiac structure and function after early childhood. In addition, not many studies describe the association between maternal obesity and offspring’s cardiac structure and function and most included studies were small and there was considerable heterogeneity amongst the included studies. Therefore, more studies assessing this association are necessary to explore the association between maternal obesity and offspring’s cardiac structure and function. However, given the high prevalence of maternal obesity and the increase in CVD risk in offspring being born after maternal obesity, it is of particular public health interest to invest in strategies to reduce obesity in women to optimize cardiovascular development and health in the next generation.

## Supporting information

S1 Fig Forest plots for cardiac structure. (DOCX)

S2 Fig Forest plots for systolic cardiac function. (DOCX)

S3 Fig Forest plots for diastolic and global cardiac function. (DOCX)

S4 Fig Adjusted Newcastle-Ottawa Scale for cohort studies. (DOCX)

S5 Fig Adjusted Newcastle-Ottawa Scale for case-control studies. (DOCX)

S6 Fig Risk of bias cohort studies. (DOCX)

S7 Fig Risk of bias case-control studies. (DOCX)

S8 Fig PRISMA 2020 for abstracts checklist. (DOCX)

S9 Fig PRISMA 2020 checklist. (DOCX)

S1 Table Search strategy. (DOCX)

S2 Table Overview measured outcomes per study. (DOCX)

S3 Table Additional measured outcomes per study. (DOCX)

S1 Data (XLSX)
