# Supplementary figures and images for: Maternal obesity in pregnancy and children’s cardiac function and structure: A systematic review and meta-analysis of evidence from human studies
Source: PLoS One. 2022 Nov 8;17(11):e0275236. doi: 10.1371/journal.pone.0275236 (PMC9642886; doi:10.1371/journal.pone.0275236)

| **S1 Fig: Forest plots for cardiac structure and dimension** |
| --- |
| 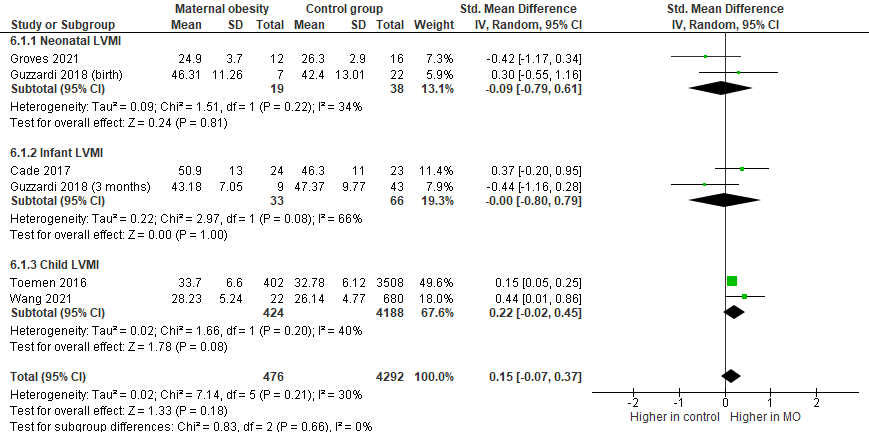 |
| 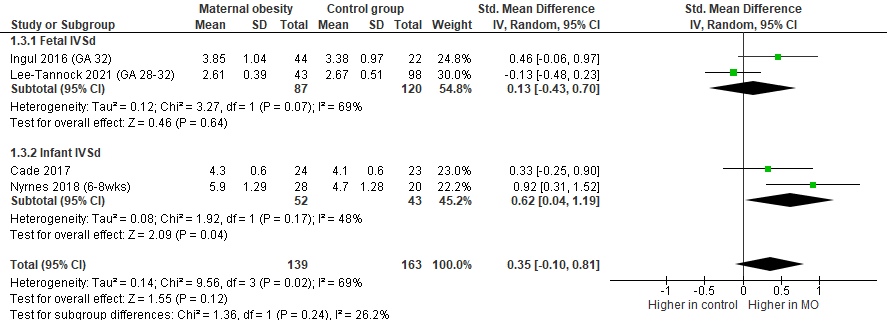 |
| 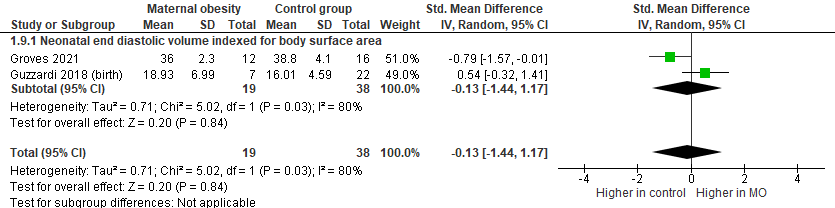 |
| 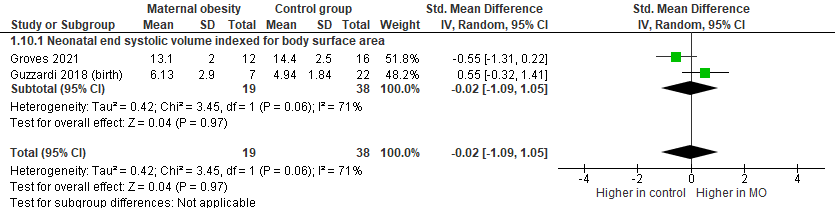 |
| 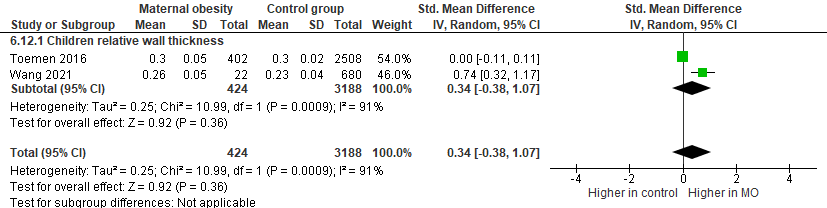 |

Supplement: S1 Fig — (DOCX) [file pone.0275236.s001.docx]

| **S2 Fig: Forest plots for systolic cardiac function** |
| --- |
| 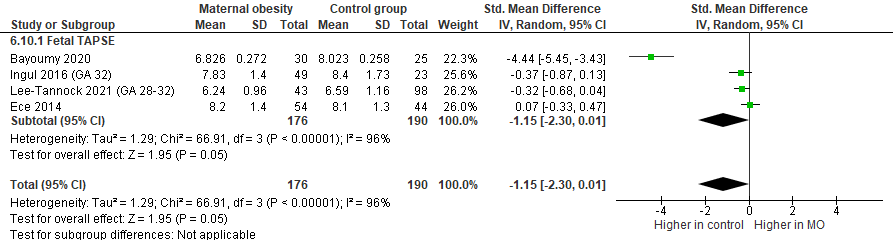 |
| 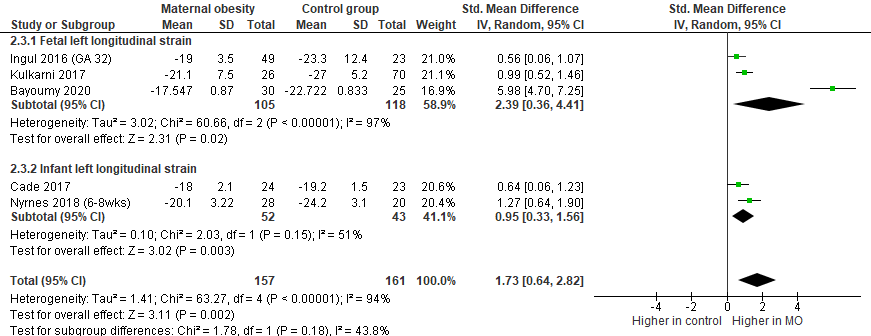 |
| 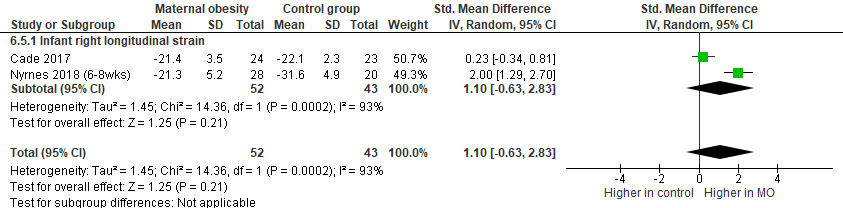 |
| 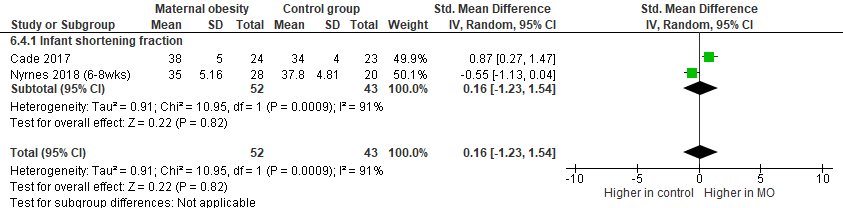 |
| 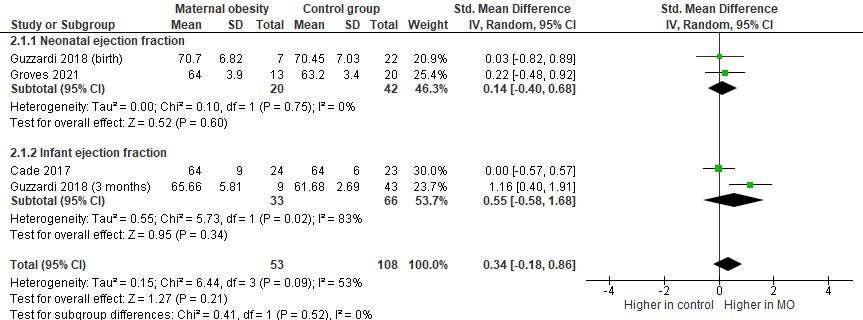 |

Supplement: S2 Fig — (DOCX) [file pone.0275236.s002.docx]

| **S3 Fig: Forest plots for diastolic and global cardiac function** |
| --- |
| 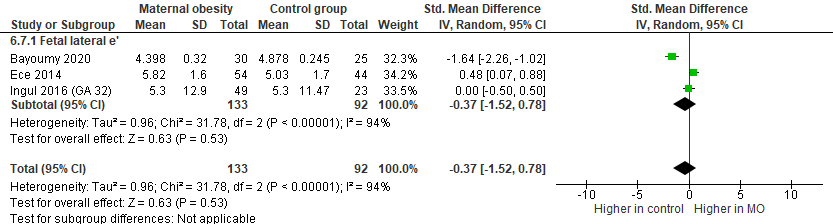 |
| 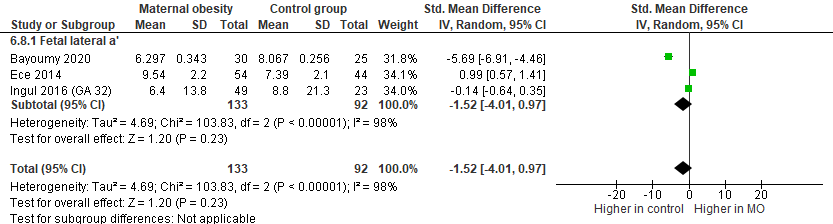 |
| 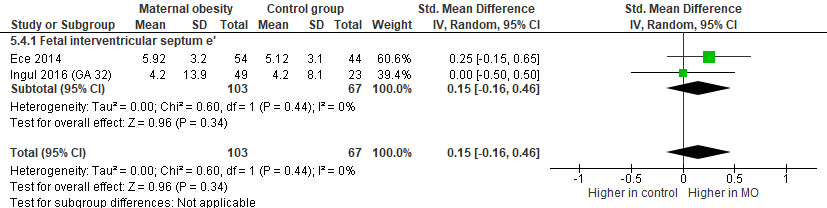 |
| 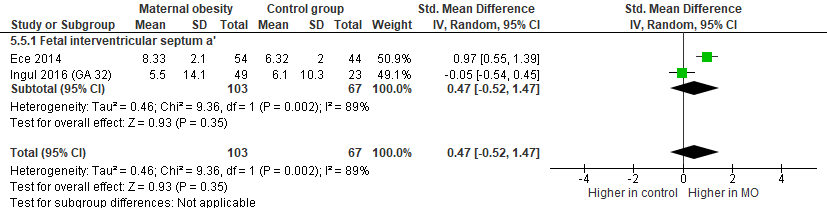 |
| 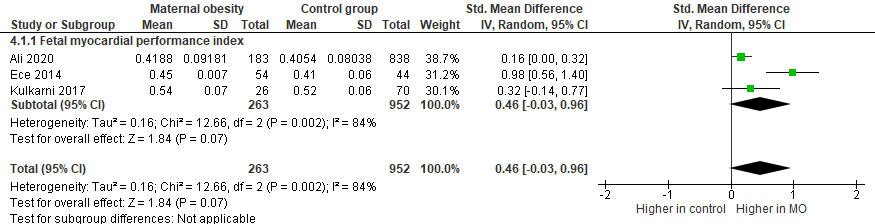 |
| 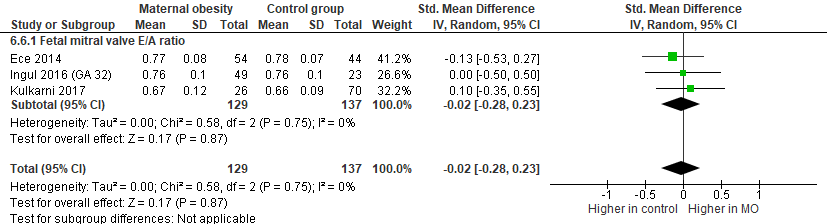 |
| 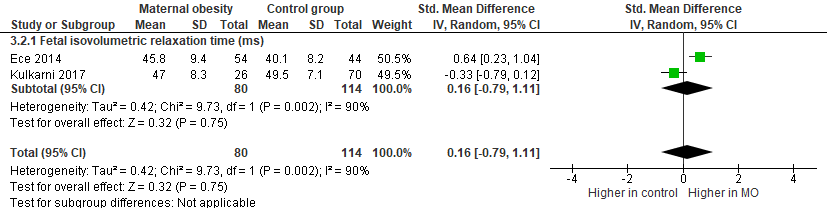 |

Supplement: S3 Fig — (DOCX) [file pone.0275236.s003.docx]

**S6 Fig: Risk of bias cohort studies**


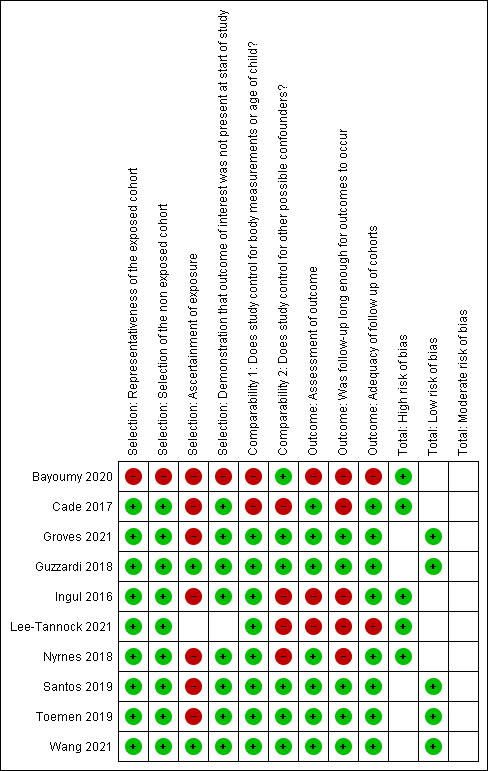

Supplement: S6 Fig — (DOCX) [file pone.0275236.s006.docx]

**S7 Fig: Risk of bias case-control studies**


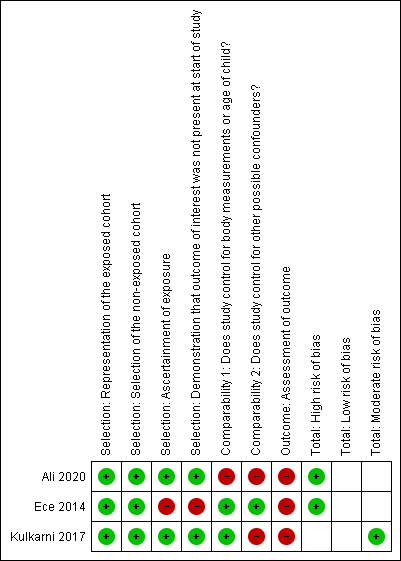

Supplement: S7 Fig — (DOCX) [file pone.0275236.s007.docx]
