# Supplementary material for: Maternal obesity in pregnancy and children’s cardiac function and structure: A systematic review and meta-analysis of evidence from human studies
Source: PLoS One. 2022 Nov 8;17(11):e0275236. doi: 10.1371/journal.pone.0275236 (PMC9642886; doi:10.1371/journal.pone.0275236)
Supplement: S4 Fig — (DOCX) [file pone.0275236.s004.docx]

S4 Fig

## NEWCASTLE - OTTAWA QUALITY ASSESSMENT SCALE COHORT STUDIES

Note: A study can be awarded a maximum of one star for each numbered item within the Selection and Outcome categories. A maximum of two stars can be given for Comparability

# Selection

1. Representativeness of the exposed cohort
   1. truly representative of the average (describe) in the community 🟑
   2. somewhat representative of the average in the community 🟑
   3. selected group of users eg nurses, volunteers
   4. no description of the derivation of the cohort
2. Selection of the non exposed cohort
   1. drawn from the same community as the exposed cohort 🟑
   2. drawn from a different source
   3. no description of the derivation of the non exposed cohort
3. Ascertainment of exposure
   1. secure record (eg surgical records) 🟑
   2. structured interview 🟑
   3. written self report
   4. no description
4. Demonstration that outcome of interest was not present at start of study
   1. yes (only applicable if ascertainment was NOT done by self report) 🟑
   2. no (if ascertainment was done by self report)

# Comparability

1. Comparability of cases and controls on the basis of the design or analysis
   1. study controls for some kind of body measurement child/age child (one of these) OR has shown these variables are NOT significantly different between groups 🟑
   2. study controls for DM mother/sex child/RR child during measurement (one of these) OR has shown these variables are NOT significantly different between groups 🟑

# Outcome

1. Assessment of outcome
   1. independent blind assessment (only applicable if measurement is done after 6 months of age OR when it cleary states analyses was done by someone different than sonographer)) 🟑
   2. record linkage (only applicable if measurement is done after 6 months of age OR when it clearly states analyses was done by someone different than sonographer) 🟑
   3. self report
   4. no description
2. Was follow-up long enough for outcomes to occur
   1. > 6 months of age 🟑
   2. <6 months of age/fetal echo
3. Adequacy of follow up of cohorts
   1. complete follow up - all subjects accounted for 🟑
   2. subjects lost to follow up unlikely to introduce bias - small number lost - > 60% follow up[1], or description provided of those lost) 🟑
   3. follow up rate <60% and no description of those lost
   4. no statement

1. Kristman V, Manno M, Cote P. Loss to follow-up in cohort studies: how much is too much? Eur J Epidemiol. 2004;19(8):751-60.
