# Supplementary material for: Maternal obesity in pregnancy and children’s cardiac function and structure: A systematic review and meta-analysis of evidence from human studies
Source: PLoS One. 2022 Nov 8;17(11):e0275236. doi: 10.1371/journal.pone.0275236 (PMC9642886; doi:10.1371/journal.pone.0275236)
Supplement: S5 Fig — (DOCX) [file pone.0275236.s005.docx]

S5 Fig

## NEWCASTLE - OTTAWA QUALITY ASSESSMENT SCALE CASE CONTROL STUDIES

Note: A study can be awarded a maximum of one star for each numbered item within the Selection and Exposure categories. A maximum of two stars can be given for Comparability.

# Selection

1. Is the case definition adequate?
   1. yes, with independent validation 🟑
   2. yes, eg record linkage or based on self reports
   3. no description
2. Representativeness of the cases
   1. consecutive or obviously representative series of cases 🟑
   2. potential for selection biases or not stated
3. Selection of Controls
   1. community controls 🟑
   2. hospital controls
   3. no description
4. Definition of Controls
   1. no history of disease (only applicable if weight/BMI was NOT measured by self report) 🟑
   2. no description of source

# Comparability

1. Comparability of cases and controls on the basis of the design or analysis
   1. study controls for some kind of body measurement child/age child (one of these) OR has shown these variables are NOT significantly different between groups 🟑
   2. study controls for DM mother/sex child/RR child during measurement (one of these) OR has shown these variables are NOT significantly different between groups 🟑

# Exposure

1. Ascertainment of exposure
   1. secure record (only applicable if measurement is done after 6 months of age OR when it clearly states analyses was done by someone different than sonographer) 🟑
   2. structured interview where blind to case/control status (only applicable if measurement is done after 6 months of age OR when it cleary states analyses was done by someone different than sonographer) 🟑
   3. interview not blinded to case/control status
   4. written self report or medical record only
   5. no description
2. Same method of ascertainment for cases and controls
   1. yes 🟑
   2. no
3. Non-Response rate
   1. same rate for both groups 🟑
   2. non respondents described

rate different and no designation
