# Supplementary material for: Maternal obesity in pregnancy and children’s cardiac function and structure: A systematic review and meta-analysis of evidence from human studies
Source: PLoS One. 2022 Nov 8;17(11):e0275236. doi: 10.1371/journal.pone.0275236 (PMC9642886; doi:10.1371/journal.pone.0275236)
Supplement: S1 Table — (DOCX) [file pone.0275236.s010.docx]

Table S1: Search strategies

Database(s): **Ovid MEDLINE(R) ALL** 1946 to September 10, 2021

Search Strategy: **2021-09-12**

| **#** | **Searches** | **Results** |
| --- | --- | --- |
| 1 | overweight/ or overnutrition/ or obesity/ or obesity, abdominal/ or obesity, morbid/ | 230331 |
| 2 | exp weight gain/ | 33599 |
| 3 | body mass index/ or skinfold thickness/ or waist-hip ratio/ or adiposity/ | 150969 |
| 4 | anthropometry/ and (mothers/ or (maternal* or mother*).ti.) | 818 |
| 5 | obes*.tw,ot,kf,jw. | 357212 |
| 6 | (adiposity or (adipose adj3 (women or adolescent* or patient* or persons or individual*))).tw,kf. | 28344 |
| 7 | (((high* or extrem* or exceed* or great*) adj4 body weight) or over-weight* or overweight*).tw,kf. | 86029 |
| 8 | (maternal size or ((maternal or mother* or preconcept* or conception or pregnan* or prepregnan* or "during gestat*" or pregestat* or gravid* or pregravid* or trimester* or prenatal* or pre-natal* or antenatal* or ante-natal*) adj2 (anthropom* or weight))).tw,kf. | 10716 |
| 9 | ((maternal or gestation* or pregestat* or pregnan* or prepregnan* or gravidit* or pregravidit* or trimester* or conception or preconcept* or antepart* or ante-part*) adj9 (weight adj2 gain*)).tw,kf. | 8312 |
| 10 | (BMI* or body mass).tw,kf. | 312131 |
| 11 | (skinfold or skin-fold or (waist adj4 (hip or hips)) or ((waist or hip) adj3 (circumfer* or girth* or height or ratio or ratios)) or WC or WHR).tw,kf. | 53698 |
| **12** | **or/1-11 [ obesity ]** | **685252** |
| 13 | (exp animals/ not humans/) or (animal* or veterinar*).jw. or exp veterinary medicine/ or exp pregnancy, animal/ or exp rodentia/ or (baboon* or pig or pigs or goat or goats* or sheep or lamb or lambs or ovine or cattle or bovine or cow or cows or horse or horses or mare or calve or calves or dog or dogs or canine or bitch* or cat or cats or feline or rodent* or rabbit* or mice or mouse or murine* or rat or rats or frog or frogs).ti. or (C57BL* or Balb-c or Balbc or wistar or  sprague-dawley or dam or dams or pups or pup or ewe or ewes or sow or sows).tw,kf. | 6010463 |
| **14** | **12 not 13 [ obesity in humans ]** | **589446** |
| 15 | fetal heart/ or heart rate, fetal/ | 13810 |
| 16 | "embryonic and fetal development"/ or fetal development/ or fetal organ maturity/ or fetal weight/ or maternal-fetal relations/ or exp heart/em | 44540 |
| 17 | prenatal exposure delayed effects/ or maternal exposure/ or prenatal nutritional physiological phenomena/ | 39775 |
| 18 | ((f?etal adj3 (origin* or start* or begin* or root* or antecedent*)) or ((development* or early) adj2 origin* adj6 (dis* or CVD)) or DoHaD* or FOAD* or FOD).tw,kf. | 5654 |
| 19 | ((f?etal or early or perinat* or peri-nat* or prenat* or pre-nat* or ante-nat* or antenat* or uter* or intrauterin* or developmental or metabolic or high-fat or cardiovascul*) adj2 programming).tw. or (programming not ICD).kf. | 8259 |
| 20 | ((f?etal or f?etus*) adj6 (card* or heart* or myocard* or ((intraventric* or ventric*) not ((intraventric* or intra-ventric*) adj2 (bleed* or h?emorr*))) or septum or aort* or tricuspid* or mitral* or atrium or atrial or ECG* or EFE or echo* or imaging or MRI or speckl*)).tw,kf. | 29737 |
| 21 | (f?etomater* or f?eto-mater*).tw,kf. | 3715 |
| 22 | ((f?etus* or offspring* or progen*) adj2 mother*).tw,kf. | 10729 |
| 23 | ((f?etus* or offspring* or progeny* or progenies or neonat* or neo-nat* or newborn* or new*- born* or girls or boys or infant* or child*) adj3 obese adj (women or mother* or pregnan* or prepregnan* or gestat* or pregestat* or gravid* or pregravid* or preconcept*)).tw,kf. | 441 |
| **24** | **or/15-23 [ A fetus / fetal heart narrow search ]** | **135301** |
| 25 | pregnancy/ or pregnant women/ or gravidity/ or exp pregnancy trimesters/ or pregnancy, multiple/ | 916949 |
| 26 | (conception or preconcept* or periconcept* or pregnan* or prepregnan* or peripregnan* or gestat* or pregestat* or perigestat* or gravid* or pregravid* or trimester* or intra-uterine or intrauterine or "in utero" or prenatal* or pre-natal* or antenatal* or ante-natal*).tw,kf. | 791739 |

| 27 | ((maternal or mother*) adj3 (obes* or OB or overweight* or over-weight*)).tw,kf. | 5472 |
| --- | --- | --- |
| **28** | **or/25-27 [ (pre) pregnancy ]** | **1180130** |
| 29 | human development/ or adolescent development/ or child development/ or puberty/ or adolescent health/ or child health/ or infant health/ | 73553 |
| 30 | exp child/ or exp infant/ or fetus/ | 2657570 |
| 31 | schools/ | 43312 |
| 32 | (offspring or progeny* or progenies or f?etus* or f?etal outcome* or babies or newborn* or new* born* or neonat* or neo-nat* or postnat* or post-nat* or girls or boys or infant* or infancy or toddler* or graders or child or childs or children or childhood or schoolchild* or school age* or schoolage* or teens or teenager* or puber* or juvenil* or youth or adolescence or adulthood or young adult* or adult life or p?ediatric).tw,kf. | 2738756 |
| **33** | **or/29-32 [offspring]** | **3803902** |
| **34** | **28 and 33 [ B pregnancy/maternal - infant ]** | **535070** |
| **35** | **24 or 34 [ A B maternal/pregnancy -infant ]** | **588900** |
| 36 | exp ventricular function/ or ventricular dysfunction/ or ventricular dysfunction, left/ or ventricular dysfunction, right/ or exp atrial function/ or myocardial contraction/ or exp coronary circulation/ | 175795 |
| 37 | heart function tests/ or cardiac output/ or stroke volume/ | 91152 |
| 38 | cardiac volume/ | 6309 |
| 39 | exp echocardiography/ | 142466 |
| 40 | fetal heart/gd, pp, ah or exp heart/dg | 63159 |
| 41 | endocardium/ or heart atria/ or exp heart septum/ or heart ventricles/ or myocardium/ or pericardium/ | 287696 |
| 42 | hypertrophy, left ventricular/ | 14522 |
| 43 | exp heart/ and organ size/ | 8951 |
| 44 | (((heart or cardiac or ventricular or ventricle* or biventric* or myocard* or septal or septum or systol* or diastol* or endsystol* or enddiastol* or hyperdynam*) adj4 (function* or d?sfunct* or malfunct* or contract* or relaxat* or performanc* or efficienc* or mechanoenerg* or mechano-energ* or programming or (TDV not vortex*))) or ((systol* or diastol* or ventricular or LV) adj3 impair*) or tissue doppler or MPI or Tei-ind* or MEEi).tw,kf. | 223954 |
| 45 | ((card* or ventric* or myocard* or concentric) adj6 remodel*).tw,kf. | 24760 |
| 46 | (cardiac output or cardiac index or (pump adj3 funct*) or ((stroke or enddiastol* or end-diastol* or endsystol* or end systol* or late diastol*) adj2 volume*) or ejection fraction* or VEF or LVEF* or RVEF or (ventric* adj3 (output or volume* or ejection* or enddiastol* or end-diast* or late-diastol* or endsystol* or end-systol*))).tw,kf. | 153226 |
| 47 | (d#ssynchron* or DYS or 2CDYS or 1CDYS).tw,kf. | 5530 |
| 48 | (((global or segment* or longitudin* or systolic or diastolic or peak* or rate or imag* or indices or index) adj4 (strain or motion* or deformat*)) or GSR or frame rate*).tw,kf. | 44940 |
| 49 | (echocardiogra* or echo-cardiogra* or ECG* or fECG* or EFE or ((echo* or imaging or MRI*) adj3 (cardiac or heart*))).tw,kf. | 240627 |
| 50 | speckle*.tw,kf. | 15723 |
| 51 | ((E-E or E-A).tw,kf. and (systol* or diastol* or veloc*).mp.) or (((E-E or E-A) adj6 ratio*) or ((diastole or diastolic or systole or systolic or veloc* or decelerat*) adj5 "E")).tw,kf. | 9341 |
| 52 | (((mitral or tricuspid* or septal or lateral) adj6 ("E" or wave* or veloc* or inflow or systol* or diastol*)) or MAPSE or TAPSE or E-vel).tw,kf. | 17178 |
| 53 | (velocity time integral* or VTI or (valv* adj3 velocit*)).tw,kf. | 2023 |
| 54 | ((systol* adj3 excurs*) or ((annulus or annular) adj3 (diamet* or plain))).tw,kf. | 3010 |
| 55 | (cardiac adj3 (structure* or shape* or diamet* or axis or size* or radius or chang* or alterat* or biometr* or developm* or workload)).tw,kf. | 31518 |
| 56 | (heart adj (structure* or shape* or diamet* or chang* or alterat* or biometr* or developm* or workload)).tw,kf. | 4835 |
| 57 | (((heart or card* or left or right) adj2 ventric*) or (LV not leg-volum*) or (RV not residual volum*) or LVOT or RVOT or LV-OT or RV-OT or outflow-tract*).tw,kf. | 280705 |
| 58 | (atrium or atria or interatr* or inter-atr*).tw,kf. | 48577 |

| 59 | (((LA or left atri*) and atria*) or lA-Ao*).tw,kf. | 36008 |
| --- | --- | --- |
| 60 | myocardium.tw,kf. | 76652 |
| 61 | ((heart or cardiac or ventr* or interventr* or atrial) adj3 sept*).tw,kf. | 43670 |
| 62 | ((f?etal or f?etus*) adj3 (myocard* or iVS)).tw,kf. | 676 |
| 63 | ((aort* adj4 (root* or central or ascend* or ejection* or strain or augmentat* ind* or AIx)) or AoV).tw,kf. | 30286 |
| 64 | (epicardium or epicardial or pericardium or pericardial or (cardiac adj2 fat) or EFT or hypo- echogen* or hypoechogen*).tw,kf. | 56291 |
| 65 | ((ventric* adj3 (wall* or cavit*)) or PWLV* or circumferential fib* or mVcf or VcF).tw,kf. | 21461 |
| 66 | (((((intraventric* or ventric* or biventr*) not ((intraventric* or ventric*) adj2 (bleed* or blood- loss* or h?emorr*))) or septal or septum or IVS or myocard* or atrium or atria*) adj6 (hypertroph* or thick* or width* or strain* or mass or masses or wall* or geometr* or circumfer* or diamet* or distanc* or dimension* or area or volum* or globul* or global or segment* or spher* or architectur* or structure* or shape* or axis or size* or radius or chang* or biometr* or developm* or workload or deformat* or lengthen* or morphometr* or morphofunct* or velocit* or motion* or TDI or ratio or ratios or index or indices)) or ((cardiac or eccentr*) adj3 hypertroph*) or LVMI or LVM or LVSI or spheri* ind* or TDI-velocit* or EDIVS or EDLVPW or relative wall thickness or RWT or LVPWd or LVID or LIVDs or LIVDd or IVSd).tw,kf. not (congenit* or defects).ti,ot. | 227653 |
| 67 | ((fraction* or midwall or ventric*) adj3 shorten*).tw,kf. | 8383 |
| **68** | **or/36-67 [ cardiac function/structure ]** | **1021938** |
| **69** | **14 and 35 and 68 [ cardiac function/structure in offspring of obese mothers]** | **462** |
| **70** | **remove duplicates from 69 [ cardiac function/structure in offspring of obese mothers]** | **462** |

Database(s): **Embase Classic+Embase** 1947 to 2021 September 10

Search Strategy: **2021-09-12**

| **#** | **Searches** | **Results** |
| --- | --- | --- |
| 1 | obesity/ or overnutrition/ or abdominal obesity/ or adolescent obesity/ or diabetic obesity/ or maternal obesity/ or morbid obesity/ | 514519 |
| 2 | exp body weight gain/ | 27388 |
| 3 | body mass/ or body fat/ or fat mass/ or hip circumference/ or waist circumference/ or waist hip ratio/ or waist to height ratio/ or weight height ratio/ or skinfold thickness/ | 568037 |
| 4 | weight chart/ or exp anthropometric device/ or bmi chart/ | 3900 |
| 5 | anthropometry/ and (mothers/ or (maternal* or mother*).ti.) | 1607 |
| 6 | obes*.tw,ot,kw,jw. | 539218 |
| 7 | (adiposity or (adipose adj3 (women or adolescent* or patient* or persons or individual*))).tw,kw. | 40806 |
| 8 | (((high* or extrem* or exceed* or great*) adj4 body weight) or over-weight* or overweight*).tw,kw. | 130732 |
| 9 | (maternal size or ((maternal or mother* or preconcept* or conception or pregnan* or prepregnan* or "during gestat*" or pregestat* or gravid* or pregravid* or trimester* or prenatal* or pre-natal* or antenatal* or ante-natal*) adj2 (anthropom* or weight))).tw,kw. | 14932 |
| 10 | ((maternal or gestation* or pregestat* or pregnan* or prepregnan* or gravidit* or pregravidit* or trimester* or conception or preconcept* or antepart* or ante-part*) adj9 (weight adj2 gain*)).tw,kw. | 11720 |
| 11 | (BMI* or body mass).tw,kw. | 546480 |
| 12 | (skinfold or skin-fold or (waist adj4 (hip or hips)) or ((waist or hip) adj3 (circumfer* or girth* or height or ratio or ratios)) or WC or WHR).tw,kw. | 81739 |
| **13** | **or/1-12 [ obesity ]** | **1183990** |
| 14 | ((animal/ or animal experiment/ or exp animal model/ or nonhuman/ or exp female animal/) not human/) or (animal* or veterinar*).jw. or exp veterinary medicine/ or exp pregnancy, animal/ or exp rodentia/ or (ape or apes or monkey* or primate* or macac* or baboon* or pig or pigs or goat or goats* or sheep or lamb or lambs or ovine or cattle or bovine or cow or cows or horse or horses or mare or calve or calves or dog or dogs or canine or bitch* or cat or cats or feline or rodent* or rabbit* or mice or mouse or murine* or rat or rats or chicken* or frog or frogs).ti. or | 8399401 |

|  | (C57BL* or Balb-c or Balbc or wistar or sprague-dawley or dam or dams or pups or pup or ewe or ewes or sow or sows).tw,kw. |  |
| --- | --- | --- |
| **15** | **13 not 14 [ obesity in humans ]** | **1043239** |
| 16 | fetus heart/ or fetus myocardium/ or *fetus heart rate/ | 11838 |
| 17 | prenatal development/ or fetus development/ or fetus weight/ or mother fetus relationship/ or fetoplacental unit/ | 85845 |
| 18 | fetus electrocardiography/ | 2196 |
| 19 | prenatal exposure/ not (congenit* or defects or anomal* or SSRI* or (serotinin* adj3 inhib*) or antipsych* or non-communicab* or immu*suppres* or toxic* or polut*).ti. | 25031 |
| 20 | ((f?etal adj3 (origin* or start* or begin* or root* or antecedent*)) or ((development* or early) adj2 origin* adj6 (dis* or CVD)) or DoHaD* or FOAD* or FOD).tw,kw. | 7756 |
| 21 | ((f?etal or early or perinat* or peri-nat* or prenat* or pre-nat* or ante-nat* or antenat* or uter* or intrauterin* or developmental or metabolic or high-fat or cardiovascul*) adj2 programming).tw. or (programming not ICD).kw. | 13025 |
| 22 | ((f?etal or f?etus*) adj6 (card* or heart* or myocard* or ((intraventric* or ventric*) not ((intraventric* or intra-ventric*) adj2 (bleed* or h?emorr*))) or septum or aort* or tricuspid* or mitral* or atrium or atrial or ECG* or EFE or echo* or imaging or MRI or speckl*)).tw,kw. | 43306 |
| 23 | (f?etomater* or f?eto-mater*).tw,kw. | 5650 |
| 24 | ((f?etus* or offspring* or progen*) adj2 mother*).tw,kw. | 15616 |
| 25 | ((f?etus* or offspring* or progeny* or progenies or neonat* or neo-nat* or newborn* or new*- born* or girls or boys or infant* or child*) adj3 obese adj (women or mother* or pregnan* or prepregnan* or gestat* or pregestat* or gravid* or pregravid* or preconcept*)).tw,kw. | 774 |
| **26** | **or/16-25 [ A fetus / fetal heart narrow search ]** | **186928** |
| 27 | pregnancy/ or first trimester pregnancy/ or second trimester pregnancy/ or third trimester pregnancy/ or exp named groups by pregnancy/ or gestation period/ or fetus outcome/ or fetus risk/ | 894681 |
| 28 | maternal obesity/ | 5593 |
| 29 | (conception or preconcept* or periconcept* or pregnan* or prepregnan* or peripregnan* or gestat* or pregestat* or perigestat* or gravid* or pregravid* or trimester* or intra-uterine or intrauterine or "in utero" or prenatal* or pre-natal* or antenatal* or ante-natal*).tw,kw. | 1083038 |
| 30 | ((maternal or mother*) adj3 (obes* or OB or overweight* or over-weight*)).tw,kw. | 8632 |
| **31** | **or/27-30 [ (pre) PREGNANCY ]** | **1368255** |
| 32 | progeny/ | 64714 |
| 33 | human development/ or exp postnatal development/ or adolescent development/ or exp childhood/ or puberty/ or adolescence/ or prepuberty/ or child growth/ or adolescent health/ or child health/ | 318180 |
| 34 | child/ or juvenile/ or exp infant/ or preschool child/ or school child/ or toddler/ or fetus/ | 3361263 |
| 35 | high school/ or kindergarten/ or middle school/ or primary school/ | 40689 |
| 36 | (offspring or progeny* or progenies or f?etus* or f?etal outcome* or babies or newborn* or new* born* or neonat* or neo-nat* or postnat* or post-nat* or girls or boys or infant* or infancy or toddler* or graders or child or childs or children or childhood or schoolchild* or school age* or schoolage* or teens or teenager* or puber* or juvenil* or youth or adolescence or adulthood or young adult* or adult life or p?ediatric).tw,kw. | 3654418 |
| **37** | **or/32-36 [offspring]** | **4750059** |
| **38** | **31 and 37 [ B pregnancy/maternal - infant ]** | **657712** |
| **39** | **26 or 38 [ A B maternal/pregnancy -infant ]** | **748194** |
| 40 | heart function/ or exp heart contraction/ or heart atrium function/ or heart left ventricle function/ or exp heart performance/ or heart right ventricle function/ or heart ventricle function/ or coronary artery blood flow/ or heart left ventricle filling/ or exp heart ventricle ejection/ or heart ventricle filling/ | 430709 |
| 41 | heart function test/ or heart output measurement/ | 9151 |
| 42 | cardiovascular parameters/ or cardiac index/ or heart afterload/ or heart left ventricle mass/ or exp heart muscle contractility/ or heart preload/ or exp heart size/ or exp heart volume/ | 147651 |

| 43 | (exp echocardiography/ or M-mode echography/ or cardiovascular magnetic resonance/) not (Turner* or anomalies or congenit*).ti. [echocardiography/ CMR ; M-mode =candidate EMTREE term] | 387861 |
| --- | --- | --- |
| 44 | exp heart ventricle/ or heart atrium/ or heart left atrium/ or heart right atrium/ or cardiac muscle/ or heart ventricle wall/ or exp heart septum/ or endocardium/ or epicardium/ or pericardium/ | 259071 |
| 45 | heart left ventricle hypertrophy/ | 39547 |
| 46 | exp heart/ and organ size/ | 1913 |
| 47 | epicardial fat/ | 1886 |
| 48 | heart development/ | 12221 |
| 49 | (((heart or cardiac or ventricular or ventricle* or biventric* or myocard* or septal or septum or systol* or diastol* or endsystol* or enddiastol* or hyperdynam*) adj4 (function* or d?sfunct* or malfunct* or contract* or relaxat* or performanc* or efficienc* or mechanoenerg* or mechano-energ* or programming or (TDV not vortex*))) or ((systol* or diastol* or ventricular or LV) adj3 impair*) or tissue doppler or MPI or Tei-ind* or MEEi).tw,kw. | 352236 |
| 50 | ((card* or ventric* or myocard* or concentric) adj6 remodel*).tw,kw. | 41695 |
| 51 | (cardiac output or cardiac index or (pump adj3 funct*) or ((stroke or enddiastol* or end-diastol* or endsystol* or end systol* or late diastol*) adj2 volume*) or ejection fraction* or VEF or LVEF* or RVEF or (ventric* adj3 (output or volume* or ejection* or enddiastol* or end-diast* or late-diastol* or endsystol* or end-systol*))).tw,kw. | 271052 |
| 52 | (d#ssynchron* or DYS or 2CDYS or 1CDYS).tw,kw. | 11235 |
| 53 | (((global or segment* or longitudin* or systolic or diastolic or peak* or rate or imag* or indices or index) adj4 (strain or motion* or deformat*)) or GSR or frame rate*).tw,kw. | 67888 |
| 54 | (echocardiogra* or echo-cardiogra* or ECG* or fECG* or EFE or ((echo* or imaging or MRI*) adj3 (cardiac or heart*))).tw,kw. | 427013 |
| 55 | speckle*.tw,kw. | 26321 |
| 56 | ((E-E or E-A).tw,kw. and (systol* or diastol* or veloc*).mp.) or (((E-E or E-A) adj6 ratio*) or ((diastole or diastolic or systole or systolic or veloc* or decelerat*) adj5 "E")).tw,kw. | 20046 |
| 57 | (((mitral or tricuspid* or septal or lateral) adj6 ("E" or wave* or veloc* or inflow or systol* or diastol*)) or MAPSE or TAPSE or E-vel).tw,kw. | 33411 |
| 58 | (velocity time integral* or VTI or (valv* adj3 velocit*)).tw,kw. | 3923 |
| 59 | ((systol* adj3 excurs*) or ((annulus or annular) adj3 (diamet* or plain))).tw,kw. | 6730 |
| 60 | (cardiac adj3 (structure* or shape* or diamet* or axis or size* or radius or chang* or alterat* or biometr* or developm* or workload)).tw,kw. | 47848 |
| 61 | (heart adj (structure* or shape* or diamet* or chang* or alterat* or biometr* or developm* or workload)).tw,kw. | 6947 |
| 62 | (((heart or card* or left or right) adj2 ventric*) or (LV not leg-volum*) or (RV not residual volum*) or LVOT or RVOT or LV-OT or RV-OT or outflow-tract*).tw,kw. | 462129 |
| 63 | (atrium or atria or interatr* or inter-atr*).tw,kw. | 78249 |
| 64 | (((LA or left atri*) and atria*) or lA-Ao*).tw,kw. | 64796 |
| 65 | myocardium.tw,kw. | 111037 |
| 66 | ((heart or cardiac or ventr* or interventr* or atrial) adj3 sept*).tw,kw. | 65456 |
| 67 | ((f?etal or f?etus*) adj3 (myocard* or iVS)).tw,kw. | 951 |
| 68 | ((aort* adj4 (root* or central or ascend* or ejection* or strain or augmentat* ind* or AIx)) or AoV).tw,kw. | 46885 |
| 69 | (epicardium or epicardial or pericardium or pericardial or (cardiac adj2 fat) or EFT or hypo- echogen* or hypoechogen*).tw,kw. | 89193 |
| 70 | ((ventric* adj3 (wall* or cavit*)) or PWLV* or circumferential fib* or mVcf or VcF).tw,kw. | 31928 |
| 71 | (((((intraventric* or ventric* or biventr*) not ((intraventric* or ventric*) adj2 (bleed* or blood- loss* or h?emorr*))) or septal or septum or IVS or myocard* or atrium or atria*) adj6 (hypertroph* or thick* or width* or strain* or mass or masses or wall* or geometr* or circumfer* or diamet* or distanc* or dimension* or area or volum* or globul* or global or segment* or spher* or architectur* or structure* or shape* or axis or size* or radius or chang* or biometr* or developm* or workload or deformat* or lengthen* or morphometr* or morphofunct* or velocit* or motion* or TDI or ratio or ratios or index or indices)) or ((cardiac or eccentr*) adj3 hypertroph*) or LVMI or LVM or LVSI or spheri* ind* or TDI-velocit* or | 360238 |

|  | EDIVS or EDLVPW or relative wall thickness or RWT or LVPWd or LVID or LIVDs or LIVDd or IVSd).tw,kw. not (congenit* or defects).ti,ot. |  |
| --- | --- | --- |
| 72 | ((fraction* or midwall or ventric*) adj3 shorten*).tw,kw. | 13531 |
| **73** | **or/40-72 [ cardiac function/structure ]** | **1506007** |
| **74** | **15 and 39 and 73 [ cardiac function/structure in offspring of obese mothers]** | **1210** |
| **75** | **remove duplicates from 74 [ cardiac function/structure in offspring of obese mothers - deduplicated ]** | **1199** |
| **76** | **75 not medline.cr. [ cardiac function/structure in offspring of obese mothers -deduplicated**  **- embase records only]** | **1109** |

## NARCIS

**NARCIS:**

**(cardiac-function* OR cardiac-structur* OR cardiovascul*) AND (maternal-obes* OR maternal-BMI)**

**3 hits**
