# Supplementary material for: Maternal obesity in pregnancy and children’s cardiac function and structure: A systematic review and meta-analysis of evidence from human studies
Source: PLoS One. 2022 Nov 8;17(11):e0275236. doi: 10.1371/journal.pone.0275236 (PMC9642886; doi:10.1371/journal.pone.0275236)
Supplement: S2 Table — (DOCX) [file pone.0275236.s011.docx]

| **Table S2: Overview measured outcomes per study** | |
| --- | --- |
| **Included study** | **Outcomes measured** |
| Ali 2020 | MPI |
| Bayoumy 2020 | GLS, LV e’, LV a’, LV s’, RV e’, RV a’, RV s’, TAPSE |
| Cade 2017 | LVM, LVMI, RWT, EF, SF, GLS LV and RV,  GLSRs LV and RV, IVS |
| Ece 2014 | IVCT, IVRT, LV MPI, MV E and A, MV, E/A ratio, TV E and A,  TV E/A ratio, MAPSE, TAPSE |
| Groves 2021 | LV EDV, LV ESV, SV, EF, CO, LVM, EDV/BSA, ESV/BSA |
| Guzzardi 2018 | LVM, LVMI, LVPWDd, EF, LV EDV, LV ESV, EDV/BSA, ESV/BSA |
| Ingul 2016 | GLS, IVS, MV E and A, MV E/A ratio, TV E and A, TVE/A ratio,  MAPSE, TAPSE |
| Kulkarni 2017 | GLS, EF, IVS, IVCT, IVRT, LVET, MPI, MV E/A ratio |
| Nyrnes 2018 | SF, GLS LV and RV, GLSRs LV and RV, IVS |
| Santos 2019 | Pericardial fat mass |
| Toemen 2016 | LVM, LVMI, RWT |
| Wang 2021 | LVMI, LVPWDd, RWT, IVSd, MV E/A ratio, EF, GLS |
| MPI= Myocardial performance index GLS= Global longitudinal strain  LVM= Left ventricle mass  LVMI= LVM index  RWT= Relative wall thickness EF= Ejection fraction  SF= Shortening fraction  LV= Left ventricle  RV= Right ventricle GLSr= GLS rate  IVS= Interventricular septum IVCT= Isovolumic contraction time IVRT= Isovolumic relaxation time MV= Mitral valve  TV= Tricuspid valve  MAPSE= Mitral annular plane systolic excursion TAPSE= Tricuspid annular plane systolic excursion  EDV= end diastolic volume  ESV= end systolic volume  SV= Stroke volume  CO= Cardiac output  BSA= Body surface area  LVPWDd= Left ventricular posterior wall end-diastolic diameter LVET= LV ejection time | |
