# Supplementary material for: Maternal obesity in pregnancy and children’s cardiac function and structure: A systematic review and meta-analysis of evidence from human studies
Source: PLoS One. 2022 Nov 8;17(11):e0275236. doi: 10.1371/journal.pone.0275236 (PMC9642886; doi:10.1371/journal.pone.0275236)
Supplement: S3 Table — (DOCX) [file pone.0275236.s012.docx]

| **Table S3: Additional measured outcomes per study**  **Data is presented as mean (standard deviation), median [range] or number (percentage)** | | | | | | | | | | | | | | | | | |
| --- | --- | --- | --- | --- | --- | --- | --- | --- | --- | --- | --- | --- | --- | --- | --- | --- | --- |
| **Developmental stage** | **Included study** | **GA at birth**  (*weeks*) | | **Birthweight**  (*kg*) | | **Females**  *(%)* | | **Maternal age**  *(years)* | | **Maternal/Offspring blood pressure** *(mmHg)* | | **Maternal/Offspring heart rate** *(bpm)* | | **Offspring anthropometrics at**  **time of measurement** | | | |
|  |  | **Control** | **Obesity** | **Control** | **Obesity** | **Control** | **Obesity** | **Control** | **Obesity** | **Control** | **Obesity** | **Control** | **Obesity** | **Control** | **Obesity** | | |
| **Fetal** | **Ali 2020** | *NA* | | *NA* | | *Unknown* | | 29.4 (5.3) | | *Unknown* | | *Unknown* | | *NA* | | | |
|  | **Bayoumy 2016** | *NA* | | *NA* | | *Unknown* | | Range 20-37 | | *Unknown* | | *Unknown* | | *NA* | | | |
|  | **Ece 2014** | *NA* | | *NA* | | *Unknown* | | 30.2  (17-  39) | 29.6  (18-  38) (NS) | *Maternal SBP*  122.1 (6.5)  *Maternal DBP*  79.4 (3.7) | *Maternal SBP*  124.3 (5.6) (NS)  *Maternal DBP*  81.9 (3.4) (NS) | *Fetal*  150.2 (16) | *Fetal*  146.3  (14.1) (NS) | *NA* | | | |
|  | **Ingul 2016** | *NA* | | *NA* | | *Unknown* | | 31.3  (4.5) | 31.4  (5.9) NS | *Maternal SBP* 109.3  (10.2) | *Maternal SBP* 123.1  (13.6) * | *Maternal*  65.7 (7.9) | *Maternal*  75.8  (12.8) * | *NA* | | | |
|  | **Kulkarni 2017** | *NA* | | *NA* | | *Unknown* | | 31  (24-39) | 30  (18-38) | *Unknown* | | *Unknown* | | *NA* | | | |
|  | **Lee- Tannock**  **2020** | *NA* | | *NA* | | 47 (48) | 25 (58) | 32 (6.1) | 32.4  (5.3) | *Unknown* | | *Unknown* | | *NA* | | | |
| **Neonatal/Infant** | **Groves 2021** | 39.9 (1.1) | 40.1 (1.1) | 3499 (466) | 3536 (428) | 24 (42,9) | 19 (61,3) | 33.0 (5.2) | 31.2 (6.3) | *Unknown* | | Asleep:  114 (9.8)  Awake:  125 (14.2) | Asleep:123 (12.6)  Awake:  134 (13.1) | *BSA:*  *0.228 (0.022)* | | *BSA:*  *0.218*  *(0.015)* | |
|  | **Cade 2017** | 38 (1) | 38 (2)  (NS) | 3.06  (0.48) | 3.32  (0.38) (NS) | 11 (48) | 12 (50) | 23(3) | 25 (5)  (NS) | *Unknown* | | 155 (17) | 160 (17) (NS) | *Unknown* | | | |
|  | **Guzzardi 2018** | 39.6 (1.3) | 39.4  (1.5) | 3.34 (0.35) | 3.40  (0.51) (NS) | 23 (53.5) | 4 (44.4) | 33.0  (4.8) | 34.1  (4.1) (NS) | *Unknown* | | *Unknown* | | *Unknown* | | | |
|  | **Nyrnes 2018** | 40.3 (1.1) | 39.4  (1.7) | 3.6 (0.5) | 3.9 (0.4)* | 11 (55) | 11 (39) | 31.2  (4.1) | 31.3  (4.6) | *Child SBP*  83 [77-  89]  *Child DBP]* 54 [49-  55) | *Child SBP*  88 [83-93]  *Child DBP*  52 [40-56] | *Child*  142 [134-  151] | *Child*  148 [141-  154] | Weight:  5.8 kg (1.1) | | | Weight:  5.7 kg (1.0) (NS) |
| **Children** | **Santos 2019** | *Unknown* | | *Unknown* | | 828  (50.5) | 86  (51.5) | 31.1  (4.7) | 30.3  (4.8) | *Unknown* | | *Unknown* | | BMI: 17.1 (2.3) | | | BMI: 20.0  (3.5) |

|  | **Toemen 2016** | 40.1 [36.0-  42.3]# | 39.9  [34.4-  42.4] | 3.4 (0.5) | 3.5 (0.6) | 1650  (49.7) | 207  (51.5) | 31.3  [19.8-  39.5] | 30.3  [20.5-  39.4] | *Unknown* | *Unknown* | BMI:16.0 (1.6) | BMI: 17.7  (2.8)* |
| --- | --- | --- | --- | --- | --- | --- | --- | --- | --- | --- | --- | --- | --- |
|  | **Wang 2021** | 39.2 (1.6) | 38.8 (2.3) | 3.5 (0.5) | 3.5 (0.5) | 351 (52) | 12 (55) | 30.9 (3.4) | 29.7 (3.5) | *Unknown* | *Unknown* | BMI  14.9 (1.5)  BSA  0.7 (0.1) | BMI  16.3 (2.7)*  BSA  0.8 (0.1)* |
| NA= not applicable NS= non-significant  BSA= body surface area  SBP= systolic blood pressure DBP= diastolic blood pressure  *p < 0.05  #= due to pooling of estimates of non-normal distributed values, the normal weight category is displayed | | | | | | | | | | | | | |
